# Designing equitable telehealth solutions for outpatient surgical care in a safety-net population: a human-centered design approach

**DOI:** 10.1186/s12913-025-12215-9

**Published:** 2025-02-12

**Authors:** Lara Z. Chehab, Diyah Mettupalli, Jenny R. Cevallos, Camille Rogine, Amanda Sammann, Sandhya Kumar

**Affiliations:** 1 Department of Surgery, University of California San Francisco, San Francisco, CA USA; 2 Department of Engineering, University of California Berkeley, Berkeley, CA USA

**Keywords:** Telehealth, Human-centered design, Underserved population, Outpatient clinic, Surgical care

## Abstract

**Background:**

The SARS CoV-2 (COVID-19) pandemic catalyzed a dramatic shift in healthcare delivery, with telemedicine emerging as a common mode of care provision. While pre-pandemic telemedicine services were more commonly used for preventive visits and had better adherence among younger and more affluent demographics, the landscape of telehealth in the post-pandemic period has shifted significantly to include surgical visits and publicly-insured patient populations. Without specific insights from patients and clinicians to guide this transition, telehealth delivery risks exacerbating disparities in access, experience and outcomes for medically underserved populations.

**Methods:**

We utilized a human-centered design (HCD) approach to gain insights into patient and clinician perspectives on telehealth delivery at a surgical outpatient clinic in an urban safety-net hospital and level 1 trauma center. During the Inspiration phase of HCD, we conducted 19 in-depth interviews with patients and surgical clinicians, and applied a combined thematic analysis and design synthesis approach to identify key insight statements representing actionable tensions across cohorts. During the Ideation phase of HCD, we held a structured brainstorming session to identify solutions and facilitated a discussion with surgical faculty to co-design and refine a prototype.

**Results:**

Interview analysis revealed 12 main themes, which were then reorganized into 5 core insights across both groups: “In-person appointments can be resource intensive for patients, making their attendance costly in more ways than one”; “When sacrificing connection for convenience, telehealth exacerbates discrimination felt by historically marginalized patients”; “Personal interactions are crucial for establishing new relationships and repairing mistrust between patients and clinicians”; “Visual cues and non-verbal communication are essential for personalized and effective surgical care”; “Patients and clinicians value the human infrastructure built into the in-person visit experience.” Brainstorming participants generated ideas from the first insight statement. Subsequent prototyping and co-design sessions led to the development of a screening prototype allowing both clinic staff and patients to book telehealth appropriate appointments.

**Conclusions:**

This study offers a HCD approach to developing insights and tailoring health service interventions to the local contexts for safety-net providers. By understanding the unique needs and preferences of underserved populations, we can develop telehealth interventions that increase adoption and ensure equitable access to care.

**Supplementary Information:**

The online version contains supplementary material available at 10.1186/s12913-025-12215-9.

## Background

The SARS CoV-2 (COVID-19) pandemic and its aftermath fundamentally changed the way that healthcare is delivered to medically underserved populations. The urgency to deliver needed care during the pandemic led to nearly 30% of all medical visits provided via telemedicine platforms, representing a twenty-three-fold [1] increase compared to pre-pandemic visits. Telehealth usage remains above pre-pandemic levels [2], particularly across surgical specialties [3, 4], indicating a normalization in the remote delivery of medical care. Healthcare settings are redesigning their care delivery structures to embrace hybrid models of remote and in-person care, taking advantage of an increased flexibility for HIPAA-compliant technologies by the Department of Health and Human Services [5]. This transition to the use of video communication platforms for clinical visits risks disproportionately benefiting more affluent, commercially-insured populations that experience fewer barriers to adopting telehealth interventions than publicly-insured, medically underserved populations [1, 6]. This is further demonstrated by the fact that telehealth use is still significantly lower among vulnerable populations [7, 8], and that, for Medicaid-insured patients, payment parity for the reimbursement of telehealth visits lags in comparison to in-person visits [9]. As such, tailoring telehealth delivery in a way that specifically increases adoption among this population is critical to address disparities and ensure equitable access and outcomes [8, 10].

Surgical care is unique among medical disciplines due to the greater role of physical exam findings in both pre- and post-operative care, where assessment of patient anatomy and wounds is crucial. Despite this, there is significant evidence that routine outpatient surgical care, particularly post-operative follow up after low-risk surgeries, is both safe and effective when delivered via telehealth platforms [11–15]. Surgical telehealth delivery has been shown to increase patient and provider satisfaction, produce cost savings, and can lead to quicker recovery times [16–18]. Despite this evidence, patient adherence to surgical telehealth visits is low [11], particularly among publicly-insured populations [7, 8] who may require additional support tailored to their resource constraints and preferences accessing telehealth platforms. While much of the prior work in surgical telehealth identifies a benefit in rural populations due to time and cost savings of eliminating travel [19], there is a dearth of insight into the specific advantages for urban safety-net patient populations.

A mixed methods study performed by our research group at the Zuckerberg San Francisco General Hospital (ZSFG) prior to the COVID-19 pandemic investigated patient experience in the surgical clinic, and found that patients face significant opportunity costs to attending their in-person appointments, sacrificing important obligations such as work and caregiving responsibilities [20]. In addition, our patients spend an average of almost 70% of their clinical appointment time waiting to be seen by their surgical clinician, which patients report worsened pre-existing mistrust for healthcare interactions and institutions [20]. While patients may benefit from the convenience and accessibility of remote care, it is crucial that telehealth interventions address specific patient and clinician expectations in order to ensure increased adoption and improved outcomes. To gain a better understanding of these expectations, our study applies the first two phases of human-centered design (HCD) − Inspiration and Ideation − elucidating key insights and describe the process of co-designing context-specific solutions for the delivery of remote surgical care among medically underserved populations at an urban safety net hospital and level I trauma center.

## Methods

### Study design & population

**Fig. 1:**
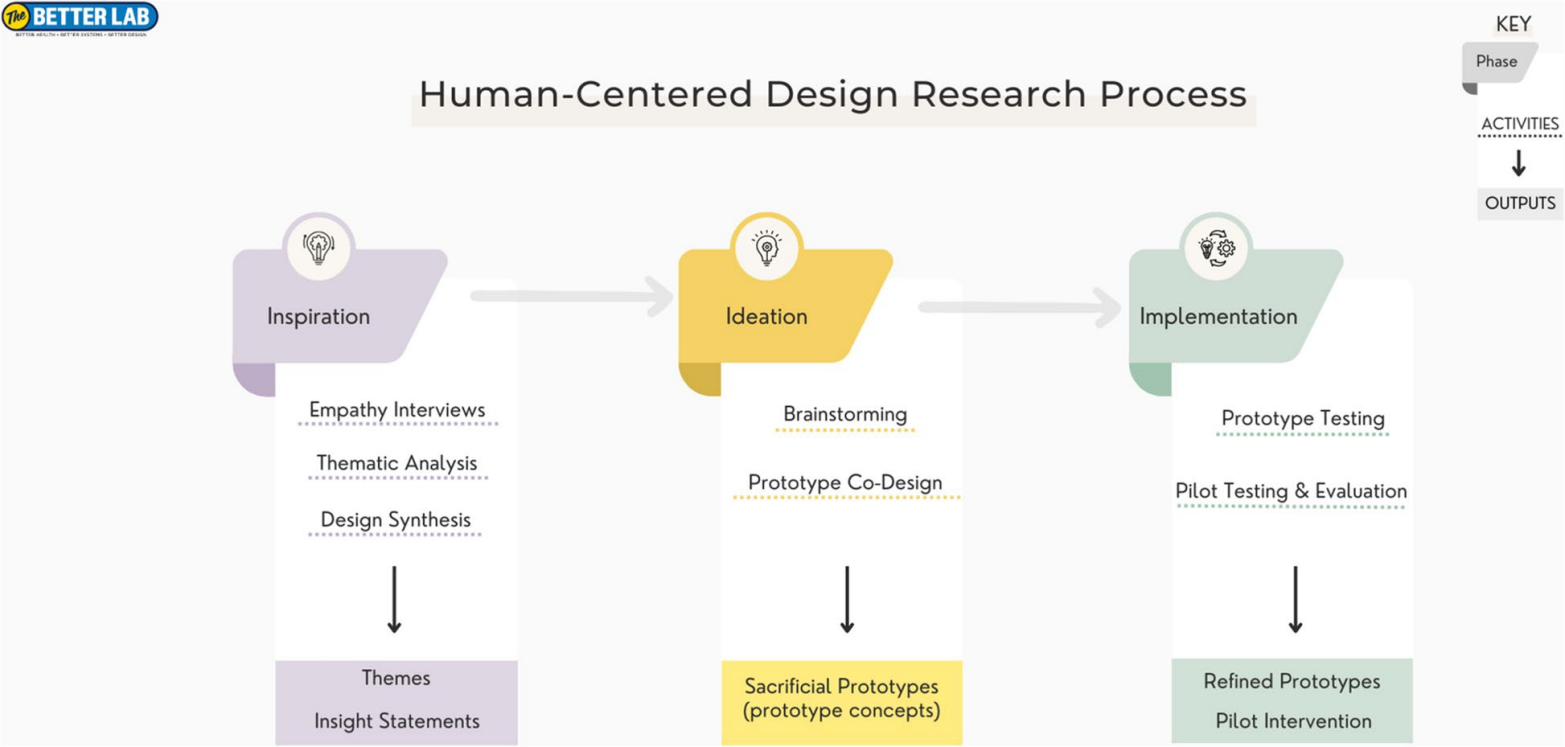
Human-centered design process and outputs

This prospective observational study adapts the first two phases of HCD, Inspiration and Ideation [21] (Fig. 1) to identify key insights specific for designing remote outpatient surgical care at the Zuckerberg San Francisco General Hospital and Trauma Center (ZSFG), an urban safety-net hospital and level I trauma center. For the year 2021 to 2022, the most recent data available, 95% of all patients receiving outpatient care at ZSFG were either publicly-insured (Medi-Cal, Medicare), subsidized by local programs or uninsured. The ZSFG outpatient surgical clinic completes about 2,000 in-person ambulatory care visits per year for general surgery and trauma care, and experiences a nearly 30% no-show rate for these visits.

HCD is a creative problem-solving approach that places users at the center and integrates their perspectives at each stage of the research process, ensuring that stakeholder needs are being prioritized in intervention design. Like other systems-based approaches, HCD considers not just the individual user but also the environment in which they operate – including the healthcare system, socioeconomic contexts and cultural norms [21, 22]. This is particularly suitable when developing or tailoring solutions for medically underserved populations whose challenges are multifaceted and extend beyond the physical healthcare setting [23]. The Inspiration phase included semi-structured interviews with patients and clinicians at an outpatient surgical clinic at ZSFG (interview guides in Supplemental Files 1 and 2), followed by brainstorming and co-design with surgical clinicians in the Ideation phase.

### Phase 1: Inspiration

The Inspiration phase leverages ethnographic research methods to identify unique unmet needs experienced by stakeholders. Interviews with patients aimed to glean insight into stakeholders’ experiences with clinic appointments and their views towards technology and telehealth. We used a convenience sampling approach to recruit patients who had appointments in the elective general surgery clinic between December 2021 and March 2022. A team of 3 design researchers (LC, DM, FC; credentials MPH, BS, BS, respectively) with training in HCD interviewing conducted interviews in English with 11 patients and 8 clinicians. Interviews lasted 30–60 min and were conducted in English, both over the phone and in-person, and compensation was a $50 gift card for their participation. Patients were asked in clinic if they would like to participate in an interview and had the option to either complete the interview in person or over the phone at a later time. Interviews used a semi-structured interview guide to gather information about how they deliver surgical care in the clinic, prior experience and comfort with telehealth, and their ideal use of telehealth. We used a purposeful sampling approach to recruit clinicians in order to ensure diversity across all surgical specialties. Clinicians were approached via email and were not compensated for their participation. Participants had no prior relationship with the facilitators and were explained the purpose of the research at the start of each interview. All interviews were audio recorded, professionally transcribed by Rev.com, and redacted to preserve anonymity.

The research team then performed a two-part thematic analysis of patient and clinician interviews consisting of inductive thematic analysis to identify emergent themes, followed by HCD synthesis to describe key insights. During thematic analysis, two researchers (LC, DM) coded a subset of interviews from each cohort to create initial patient and clinician codebooks, which were subsequently refined during consensus-building discussions between the two researchers. Once consensus on the codebooks was achieved, the researchers coded the remaining transcripts. They reviewed the quotes organized by codes, and consolidated related or redundant codes in order to develop a set of unique themes for each cohort. During HCD analysis, the researchers facilitated a discussion about the themes with the broader research team, including design researchers and clinicians providing care to surgical patients. The purpose of this discussion was to discuss thematic saturation and develop insight statements, which identify tensions or synergies between the thematic findings to represent user perspectives and motivations that describe a human need [20].

### Phase 2: Ideation

The Ideation phase builds upon a core set of insights in the Inspiration phase to develop design opportunities [24] and associated solution concepts, or “sacrificial prototypes” [25], for stakeholder testing. A brainstorming session was held with design researchers and clinicians, where participants generated solution ideas in response to brainstorming prompts, or “How Might We?” (HMW) statements inspired by the HCD insights. Ideas were organized thematically, and participants voted on their top three ideas based on perceived desirability, feasibility and impact towards delivering equitable telehealth solutions. The idea that was ranked the highest by all participants was selected for prototype development for subsequent testing with clinicians who deliver surgical care in the clinic. A visual representation of the idea was developed and presented to a group of 8 surgical faculty members, and a discussion was facilitated to gather feedback and co-design improvements to the prototype. Subsequent iterations were made to the prototype to inform initial and upcoming testing in the clinic.

## Results

The median age of patients interviewed was 51 years old (range: 28—69). Six patients identified as male, 4 identified as female and 1 identified as transgender female. Roughly half of patients identified as White (n = 6), 2 patients identified as Hispanic, 1 as Black and 1 as Other. One patient reported experiencing food insecurity, 3 people were at risk for substance use and 1 person was at risk for intimate partner violence. Clinicians interviewed included 6 attending surgeons (3 general surgery, 2 trauma surgery and 1 vascular surgery), 1 nurse practitioner (trauma surgery) and 1 physician’s assistant (vascular surgery.)

### Phase 1: Inspiration

Thematic analysis of interviews produced a set of 12 total themes, 6 for the patient cohort and 6 for the clinician cohort. HCD synthesis produced 5 main insights across both cohorts. Each insight statement is followed by a description of patients’ and clinicians’ perspectives, including exemplary quotes from the interviews. A full description of insights, themes and quotes can be found in Table 1.

**Table 1:** Insights, themes and representative quotes from patient and clinician interviews

Insight 1: In-person appointments can be costly for patients	
Patient theme 1: appointment access	“I mean, if there’s nothing wrong with you, great. You know, six months down the line is fantastic. But when you’re battling something, and now you’ve got to wait four months to see a doctor, that’s kind of what I’m talking about. You know, not having the ability to get in there when you really need to.”—Patient 3
	“It actually took me a very long time to get that appointment. Because they explained it to me, due to COVID-19, it was very hard to appointment, because it was a long line of patients waiting for that sort of appointment. So, yes, I waited for a long time.”—Patient 4
	“It would just like, “Yeah. This person can see you in three and a half weeks.” I’m like, it kind of defeats the purpose of it being convenient.”—Patient 7
Patient theme 2: getting to clinic	“It’s just, long story short, it’s the distance to get there. I live up on California Street, and learning the new system, I’m relearning it, it’s tough. There’s not enough money for an Uber, but it’s just the distance. It’s out on the trail, so to get there you have to plan enough time to do that. So, an hour there and an hour back just in travel time”—Patient 1
	“If I lived closer to the hospital, I wouldn’t mind going in person. But because I am quite a distance away, I just like doing it over the phone, it makes it easier and for me to be there on time.”—Patient 5
	“I was late but I got a 15 min grace period from 9:30. But I had to take the T rail and then get off to catch the 48. Because the 29 left me. And then it would’ve dropped me to the 9 and the 9 would’ve had me here. So, and you don’t know. It’s iffy. Because if something happens on the bus, then you’re screwed.”—Patient 8
	“Yes. Sometimes. Yeah. They take me a lot because then I have two girls and I don’t have time sometimes. And I need to take a little bit more time to come in here. And, I’m not driving. Because now with the pandemic, it’s a little bit hard, because my daughter she’s 20 months and she doesn’t use the mask and I’m a little bit scared with her.”—Patient 9
Clinician theme 1: appointment access	“But I think nonsurgical patients, post-op patients or surveillance patients, check-ins, answer questions. Those are really, I think, well suited, and especially if they’re from a remote area. I mean, if they’re in the neighborhood, but it’s hard for them to get on the bus and it takes all day. It’s just easier just to do a video visit.”—Vascular Surgery Physician
	“I think it’s better for patients, with the population that we have here, we have a lot of underserved and homeless and elderly, transportation for them is a big factor for attending. So [telehealth is] helpful for them.”—Trauma Surgery Nurse Practitioner
	“I think that in general they probably would like a video visit because they don’t have to figure out how to get here, pay for parking, go through all those different barriers, especially when we’re off time. Because of the way this clinic is scheduled, so their visit is at 9:30 and we had four patients scheduled at nine, it’d take me to 10. And so their visit doesn’t actually start until 10 or 10:15. So they’ve been sitting around waiting. I also would probably feel a little bit more comfortable if this is a video visit, honestly, of being like, “I’m sorry, we’re at the end of our half hour. I have another video visit call,” because they haven’t trucked their way in.”—Trauma Surgery Physician
	“Especially if they don’t have the means. They’ve figured out childcare for the day, gotten on a bus to come across town, taken their whole day to come over here and they get you to come back again or we didn’t have time or the doctor’s running late or there was an emergency and the visit got canceled.”—Trauma Surgery Physician
	“If a patient’s coming from a long way away, it just helps alleviate that. Over there, I have patients coming from rural parts of California and it saves them a whole travel day just to be seen.”—Vascular Surgery Physician
	“Well, it’s certainly an efficient way to deliver care in some ways and for certain patients where it’s too much of a hardship for them to come, it’s appropriate.”—General Surgery Physician
	“So distance wise they’re not as challenged, although in terms of work and family care, maybe they are. But there was one example where I had a hernia patient and I was trying to call him because he was going to come from like two hours away, so I was going to try to call him to just do it over the phone because I would feel bad for him to come just to say hi for five minutes and take two hours to drive, like take basically half a day.”—General Surgery Physician
	“It would be another mechanism of at least talking or seeing a patient that has lots of social issues. These dialysis patients seem to be the worst, but probably because they have to go somewhere three times a week. They rely on rides. After dialysis they’re tired, they don’t want to do anything. So it would just be another modality of catching some of these hard to catch patients.”—Vascular Surgery Physician’s Assistant
Insight 2: When sacrificing connection for convenience, telehealth exacerbates discrimination felt by historically marginalized patients	
Patient theme 3: patient mistrust	“And then he ordered some labs for me, and then when I go that day, he said, “Oh, you’re not… You don’t have any labs ordered.” And I said, “That’s the trigger for me.” So I was like, “Okay, forget this. What I’m going to do, I’m going to find a way around him for this.” Because you’re not going to sabotage my up and coming [surgery], because now I got to wait two months anyway, to get this stuff. This was last year.”—Patient 8
	“Some of them I do [trust]. But no, because it depends on what they do. I trust [my doctor]. I don’t trust the other ones. Hell no.”—Patient 3
	“It’s like they got a network that goes against me. They always come back and say, “Oh, you don’t have no appointment in the computer.” And I’m like, “I do have appointment in the computer.” “Well go home and get the paperwork.” And I was like, “Dude, are you serious?” So, having a hernia, that’s stressful and it makes you kind of upset.”—Patient 1
	Some of them, they just don’t listen. They hear, but they don’t listen. And sometimes you need to show something, and you cannot, actually.”—Patient 4
Clinician theme 2: patient mistrust	“They either seem, they’re like, I don’t know what the word is, but there’s a few patients I’ve seen that sometimes are very like reticent to talk in the first place, and that it’s hard to know through a second language if it’s like just institutional distrust or just nervous or overwhelmed. Could be many of those. I suspected some of those things. And those are the hardest ones because I feel like I’m not sure if they’re understanding what I’m saying and I’m not sure if I’m hearing everything they want to say to me either.”—General Surgery Physician 1
	“If you do that you’re going to encourage patients to see the interaction in a more transactional manner, as more of a commodity rather than something a little more personal. So you have undermined what is a personal relationship into a transactional form. “—General Surgery Physician 1
	“One thing I just wonder about, does it help or not help their institutional trust… And I don’t know if they see it as we’re offering more advanced things or that we’re actually shortchanging them on care. Which I think comes down to giving everyone the choice.”- General Surgery Physician 3
	“When I called him, he’d already left early and was actually in the parking lot, and so we had a face to face just for a postop visit. And basically he was like, this is such a big difference from a small operation with regard to how much better his life is and how the pain has gone and everything is so much better. And I was thinking to myself that I’m so glad we had the face to face and he was glad too, because it’s not just the doctor being selfish and wanting to see patients, the patients also feel fulfilled in the sense of being able to convey their gratitude and it’s more than just, Hey, you’re getting paid for this, thank you for providing this versus you’re actually helping out.”- General Surgery Physician 2
	“There’s a doctor’s point of view, but there’s a patient’s point of view, the way we organize it and the way we promote it, if we promote it as a commodity, then the patients are going to start to view things differently too. So it’s a two-way thing, it comes from both directions.”- General Surgery Physician 1
Insight 3: Personal interactions are crucial for establishing new relationships and repairing mistrust between patients and clinicians	
Patient theme 4: patient—clinician relationship	“My very first phone appointment was with a doctor that I had never met from the hospital. I’d never met him, and his phone appointment was okay, but I had never met the man, didn’t know anything. He never ended up being my doctor, which was really weird, because everybody said he was my doctor, but I’ve never ever seen the man”—Patient 3
	“She’s so very nice with me and I feel so happy because I’m coming here. I was a little sad, but when I talk with her now, I feel so happy.”—Patient 9
	“When it’s the first time in a clinic, it’s very hard to explain everything. Some of them, they just don’t listen. They hear, but they don’t listen. And sometimes you need to show something, and you cannot, actually.”—Patient 4
Clinician theme 3: patient-clinician relationships	“And video as an adjunct, in terms of a more developed relationship, like people that you have done a big operation on, that you’ve seen in the hospital, that you’ve known for a while, that is probably less harmful in that setting because you know them, so it’s not going to influence the interaction as much, but for patients that you see only once or twice, or for smaller procedures like inguinal hernia repairs, which I do at the VA, not here, but for the proctology patients, it’s probably likely to impact those patients more”—General Surgery Physician 2
	“It’s that patient that comes in and tells you in five minutes that you’ve changed their lives, for us, it’s a routine thing, but for that patient it’s not, and to be reminded of it actually is very, very helpful for a doctor.”- General Surgery Physician 1
Insight 4: Visual cues and non-verbal communication are essential for personalized and effective surgical care	
Patient theme 4: patient—clinician relationship	The personal contact, I guess. The being able to actually look into somebody’s eyes and watch their mouth move…That kind of thing going, “Okay. Their ears are listening to what I have to say,” and “Okay, she looks neat, or he looks neat, and] this is going well,” that kind of thing, just the personal contact of it.”—Patient 4
	“I love coming to the appointments here because the doctor’s very kind. Very helpful. They help me out a lot. They figure out what the problem is. What’s not the problem is. And they walk me through it and they don’t make me nervous. They make me feel comfortable.”—Patient 10
	“I would like to see her [on video] and say, “Hey, how are you doing?” And all that stuff. Yeah. Because I already know her.”—Patient 3
	“If I had something that was like a skin lesion or something like that, then a video would seem more appropriate and I would be comfortable doing that. If it was something that you just look at, the first thing you can just look at, then a video would be perfectly fine with me.”—Patient 7
	“But if I was able to get up, and they wanted me to come in after the surgery, I would definitely feel better going to see her then, if she had to see a wound or something like that was there, that would be okay.”—Patient 3
Patient theme 5: familiarity with technology	“We’ve all gotten used to these phone appointments now, each and every one of us around the world’s gotten used to those because of the COVID and because of this and that.”—Patient 3
	“I talk to my daughters in Pennsylvania, and, once in a while, we video chat from PA, and I video chat with my grandson from Washington, and he does all these weird little faces and being an astronaut floating up in the air.”—Patient 3
	“FaceTime’s great. Zoom, all those other, all are great. But I love the online portals that the hospitals all have now, on Myhealthcare.com, that shows you all your appointments and what you’ve done, and all your COVID vaccinations and everything else, and the other vaccinations. That’s kind of cool, that technology really helps”—Patient 1
	“I use technology every day on my phone, most of the day anyways”—Patient 5
	“I use [technology] every day, like online school, phone. All that stuff. Zooming, a lot of things for work I do remote. So a lot of tech stuff.”—Patient 7
Clinician theme 4: clinical assessment	“There is some data that you get by just physically looking at someone. You may not be able to tell all them on video, but some of it you will, like how quickly they move, do they look well taken care of, do they look well groomed, can they get in a chair and out quickly or in normal amount of time, or can they barely get out of the the or the chair to the exam table? I think you’ll be able to tell some of those even on video. It’s adding to my gestalt about the patient to be able to see them. And I think for the patient too, I can only guess because I don’t know, but to see their surgeon at some point, visually is probably important to them is my guess.”—General Surgery Physician 3
	“If I’m having a conversation about like, “Hey, we’re going to have surgery next week…I want to see them.** I** want to be able to look at them in the eyes and be like, or whatever, look them in the virtual eyes and be like, you’re listening to me. I can tell you’re listening. You’re not doing something else and going uh-huh. Yeah.””—Trauma Physician
	“There’s something about being able to see someone that improves personal connection for one. And then two, I may pick up on subtle cues that I haven’t otherwise, especially if I haven’t seen somebody for a while. So if I haven’t seen somebody for a while, especially somebody that we’ve been working toward weight loss for something, and I see them on the video and I’m like, they look like they have put on substantial weight. You know what? I think we should see each other in person before we finalize the schedule. And sometimes I’ll pick up on other subtle cues of they’re not doing well, especially if it’s a post-op follow up visit. I can tell like, you look like you’re not feeling well. You look like you’re tired. It may prompt me to ask some questions I may not otherwise. […] If we weren’t going to do routine labs and I might not pick up if I was just talking to them on the phone.”—Trauma Physician
Insight 5: Patients and clinicians value the human infrastructure built into the in-person visit experience	
Patient theme 6: feeling taken care of	“Well, the appointment at the clinic was just great. I mean, I think that hospital really does a good job of taking care of their patients. I really do. I feel that, every step of the way, they take care of you. Registration, they take care of you when you’re waiting to go in. They keep asking you, you know, “Have you been taken care of?” Things like that? And then, when you get to see your doctor, they’re really on top things.”—Patient 3
	“It was good actually this time here. I mean, it was pretty quick, because my appointment was for nine o’clock, and I got there 8:30, 8 o’clock or so. And I had to wait an hour, but I knew that. But at five minutes before 9:00, I had to go to the bathroom. So I went and did that and I come out and they were calling me. I was like, “Oh wow.”—Patient 2
	“Yes. And now the two days now send me for remember they have appointment. It’s good. They help me alot because sometimes, and I have the mother of two girls and I don’t know exactly the time. I know the date, but not the time.”—Patient 9
Clinician theme 6: the telehealth clinic	“I would go to clinic, sit down with a headset, have my video visit appointments. Also that way, the nice thing about being in clinic when I do that, is that if there are things that need to happen, like, hey, we need to schedule for this. I could ask our NP and support people in clinic. It would be lovely if I could be, “I’m going to put Mrs. Jones on hold. Can you pick up on this and get her scheduled for this, this, this, and this,” for her, as opposed to me writing this all down from clinic and I’m emailing Helen at the end of it, and then having a follow up that she actually received my message and that sort of stuff”- Trauma Surgery Physician
	“It’s nice to have a place to do it that’s not like a group workroom. I don’t know how people are doing it, but it’s nice just to have a private place to have a private conversation with somebody where the people came over here. I know my office doesn’t have a webcam or a microphone. I’m using my personal laptop. You’d just take a clinic room. Every clinic room computer would have its own webcam and microphones, and then you do it that way.”- Vascular Surgery Physician
	“The last thing is that just it’s seamless, that our MAs check the patients in, they do the thing that they do, which is like review the meds and they ask their screening questions. And then they let us know, okay, the patient’s ready. And then you just maybe walk in the room so you have a private place, like walk in the exam room that’s set up for telehealth, and then I do my visit, and I don’t really need to do any extra things. And then finally I think that I would, the translation part needs to be really well integrated, that we’re not trying to call like…”—General Surgery Physician 3
	“ “Hey, I’ve got a nine and a 9:15 video visit. I’ve got a 9:30 in person. I’ve got a 10 and a 10:15 video visit. I’ve got a 10:30.” I could definitely see a hybrid, especially what I think would happen is identify a few people from video. I need this person to come in because I really need to have an examination of them before we decide what we’re doing”- Trauma Surgery Physician

#### Insight 1

In-person appointments can be resource intensive for patients, making their attendance costly in more ways than one.

#### Description

Patients report having to make sacrifices in their daily lives in order to attend their appointments in the clinic. Patients often have to compromise caregiving responsibilities and hourly work obligations, which are further compounded by transportation challenges during their journey to the hospital. One patient said *“I have two girls and I don’t have time, but I need to take a little bit more time to come in here. And I’m not driving. It’s hard because my daughter she’s 20 months.”*; another noted that *“there’s not enough money for an Uber, it’s just the distance. To get there you have to plan enough time to do that, so an hour there and an hour back just in travel time.”* Clinicians, who provide outpatient as well as inpatient and emergency care in the hospital, describe the impact of this division of responsibilities on patients in clinic: *“They’ve figured out childcare for the day, gotten on a bus to come across town, taken their whole day to come over here and the doctor’s running late or there was an emergency and the visit got canceled.”* Both felt that having the option for a telehealth visit could mitigate the high opportunity cost for patients by improving access and reducing the burden associated with attending appointments in the surgical clinic.

#### Insight 2

When sacrificing connection for convenience, telehealth exacerbates discrimination felt by historically marginalized patients.

#### Description

Despite potential improvements in access, medically underserved patients are wary that telehealth use will further exacerbate experienced discrimination and de-prioritization of their healthcare. At baseline, patients are skeptical of healthcare personnel and are defensive of new systems and technology that have the potential of sacrificing quality of care. *“Some of them, they just don’t listen. They hear, but they don’t listen. Sometimes you need to show something and you cannot over telehealth.”* Clinicians also felt sensitive to their patients feeling commoditized as a result of using telehealth: *“If you do telehealth, you’re going to encourage patients to see the interaction in a more transactional manner, as more of a commodity rather than something a little more personal. [It] undermines what is a personal relationship into a transactional form.”*

#### Insight 3

Personal interactions are crucial for establishing new relationships and repairing mistrust between patients and clinicians.

#### Description

Patients place disproportionately higher value on the first interaction with their clinician compared to other appointments. Medically underserved populations approach new healthcare interactions with caution and prefer establishing relationships in person, rather than using telehealth to establish a new relationship. One patient noted that *“my very first phone appointment with a doctor was okay, but I had never met the man, he didn’t know anything. He never ended up being my doctor, which was really weird because everybody said he was my doctor, but I’ve never even seen the man.”* Clinicians also prefer establishing a relationship in person, and need to do a comprehensive physical exam which they need to perform in person. A general surgery attending physician explained that *“in terms of a more developed relationship, like people that you have done a big operation on, that you’ve seen in the hospital, that you’ve known for a while, telehealth is probably less harmful in that setting because you know them.”* Both felt that at least one in-person interaction prior to performing surgery was important to establish familiarity and mutual trust.

#### Insight 4

Visual cues and non-verbal communication are essential for personalized and effective surgical care.

#### Description

Patients and clinicians both appreciate the value of video capabilities during telehealth visits to enable them to use non-verbal cues to communicate with one another. Patients describe using visual cues from their provider to evaluate if they are being heard: *“The personal contact. The being able to actually look into somebody’s eyes and watch their mouth move. Their ears are listening to what I have to say.”* Clinicians also report wanting video in order to visually connect with the patient, and to assess patient understanding and frailty. *“There’s some data that you get by just physically looking at someone. You may not be able to tell everything on video, but some of it you will. Like how quickly they move, do they look well taken care of, can they get in a chair and out quickly? It’s adding to my gestalt about the patient to be able to see them.”* Clinicians also described certain visit types as not being clinically appropriate for telehealth, as they might require physical exams and in-person visual examinations. Patient participants anticipated some challenges with the technology aspect of video visits, but felt confident that they could learn given their familiarity with online platforms used for healthcare and video calling in their personal lives. *“We’ve all gotten used to these phone appointments now, each and every one of us around the world’s gotten used to those because of the COVID and because of this and that.”*

#### Insight 5

Patients and clinicians value the human infrastructure built into the in-person visit experience.

#### Description

Telehealth is most desirable by both patients and clinicians when the visit mimics a team-based workflow similar to in-person visits. Patients value feeling taken care of by a team of clinicians and staff from check-in to check-out, and don’t want that sacrificed during a telehealth visit. *“They really do a good job taking care of patients. I feel that every step of the way, they take care of you. Registration, they take care of you when you’re waiting to go in. And then when you go in to see your doctor, they’re really on top of things.”* Clinicians and staff also report wanting to maintain similar workflows to in-person visits, so as not to create added work and foster a team environment. *“It’s just seamless. Our MAs check in the patients, they do the thing that they do, which is review the meds and ask their screening questions. And then they let us know, okay the patient’s ready. And then you just walk into the exam room that’s set up for telehealth, and then I do my visit and I don’t really need to do any extra things.”*

### Phase 2: Ideation

The Ideation phase described in this study demonstrates the process of brainstorming, solution development and concept testing for the first insight: “In-person appointments are costly for patients.” Discussion sessions with researchers and clinicians around this access-related insight led to the development of the following brainstorming prompt: “How might we improve access to the clinic by empowering patients and clinicians to select telehealth appropriate appointments?” In the brainstorming session, participants generated 53 distinct ideas across 10 different themes. After categorizing and consolidating ideas, participants voted and identified their top idea for initial testing in the clinic, which was a clinician-facing screening tool that would enable clinicians and staff to identify patients who are clinically suitable for remote visits and offer them a telehealth option prior to their appointment.

**Fig. 2:**
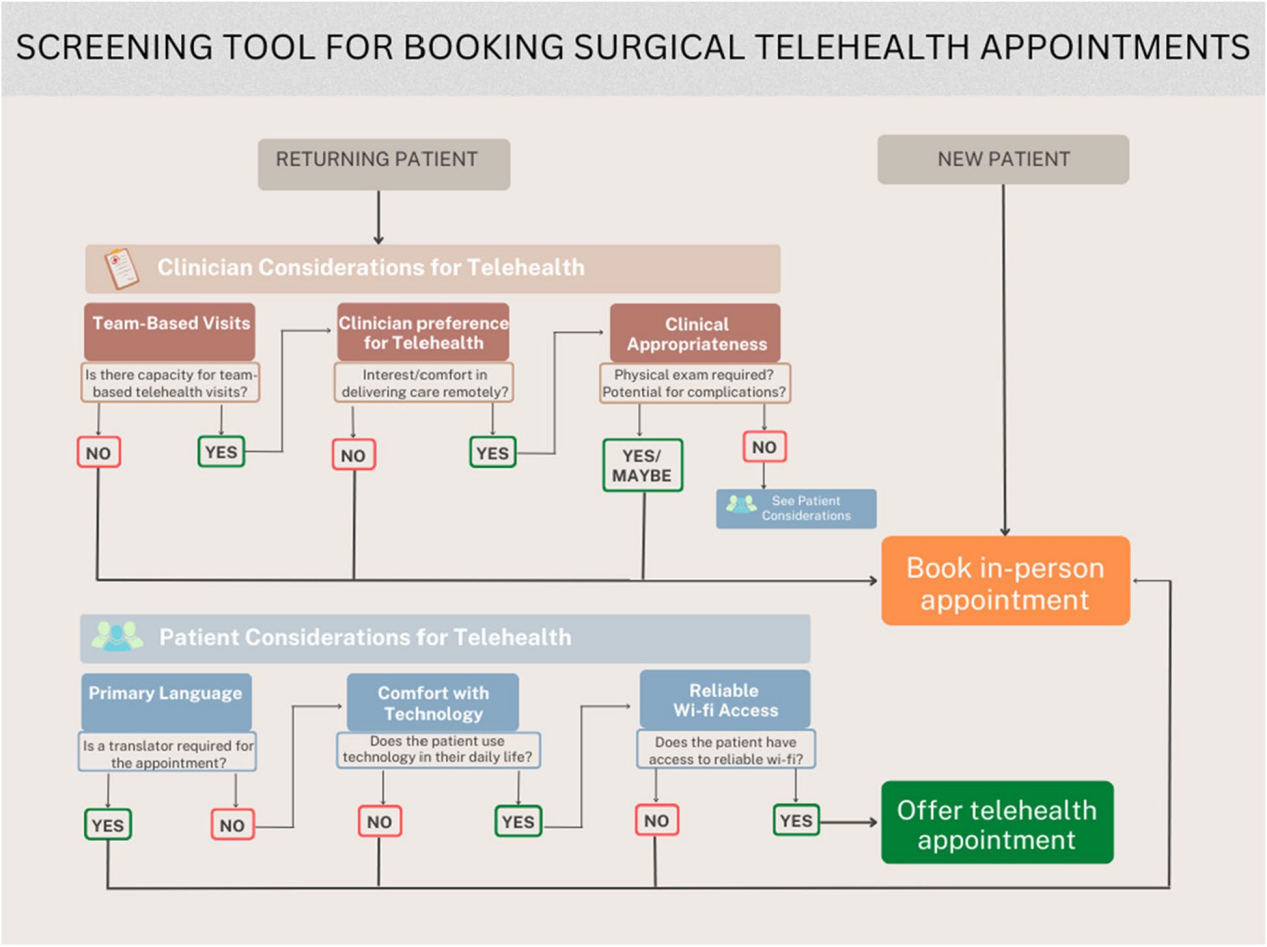
Sacrificial prototype: screening workflow

This idea was then translated into a visual prototype by the research team (Fig. 2), and incorporated relevant aspects of the other insights. As described in insight 3, establishing new relationships with patients requires personal connection and can set the tone for subsequent care. Therefore, the screening tool ensured that new patients (e.g. referrals to the clinic) were immediately booked for an in-person appointment, whereas patients who had previously established care in the clinic (e.g. follow-up patients) would be offered telehealth as a visit option. The screening tool then divided items into clinician- and patient-facing considerations in order to determine whether a telehealth appointment might be clinically appropriate, technologically feasible and acceptable by both clinicians and patients. Clinician-facing considerations were built upon findings from the interviews, including capacity for ‘team-based visits’ (insight 5) and clinical appropriateness (insight 4). Patient-facing considerations were based mostly on technological feasibility, including the patient’s comfort with technology and their Wi-Fi access. As indicated in insight 2, patients want to avoid losing the in-person connections needed to develop trusting relationships. As such, the screening tool frames telehealth as an option for their visit, empowering patients to choose between seeing their clinician in person or remotely. Several changes were made to the screening tool based on feedback from surgical faculty members, including whether clinicians are comfortable delivering the same level of care remotely as they would in person for a particular patient, and the clinic’s current inability to provide high quality, person-centered translation services that could be integrated into telehealth platforms. These two items were added to the clinician-facing considerations category (‘Clinician Preference for Telehealth’) and to the patient-facing considerations category (‘Primary Language’).

## Discussion

This study leveraged the rigor of thematic analysis and the creativity of HCD to determine key insights and potential solutions for delivering remote surgical care at an urban safety-net hospital outpatient clinic. By combining these two complementary approaches, we were able to identify and describe unique local needs and potential solutions. In this study, we identified 5 robust insights that represent the needs of both patients and clinicians when designing, developing and implementing a system of remote care for medically underserved patients. While the findings themselves described in this study are not necessarily generalizable to other surgical safety-net clinics with different populations and needs, they may offer principles for designing remote care solutions that could be transferable to other settings. Telehealth solutions must be tailored to patient preferences and their unique contexts, as perceptions have been found to vary among urban safety-net populations [26].

The insights revealed that telehealth solutions must be tailored in a way that benefits stakeholder access and convenience while prioritizing the human experience of providing and receiving surgical care. Insight 1 speaks to how telehealth can help to offset the opportunity cost for patients when attending in person appointments in the clinic. Patients cited time, childcare costs, and transportation as factors that contributed to the overall ‘cost’ of a clinic visit. This is consistent with previous work at our own institution showing that patients not only lack affordable and accessible transportation, but they also struggle to find the support necessary to attend their appointment, leading to non-attendance and increased socioeconomic stressors [20]. Insights 2 and 3 demonstrate that the relationship between patient and surgeon is crucially important for the establishment of trust, which in turn supports the delivery of effective surgical care. Telehealth solutions must therefore be mindful of how this relationship might be compromised during remote visits, particularly among a patient population with baseline mistrust for the medical system. Insights 4 and 5 reveal that both patients and clinicians want telehealth visits that come as close as possible to mimicking the in-person visit experience. When prioritizing which prototypes to test, our research team used results from the brainstorming session to identify that patient screening and selection was a necessary first step to designing subsequent telehealth solutions for the surgical clinic.

There were several limitations to this study, the first of which includes the absence of non-English speaking patients in our interviews. This is an important limitation given that only 53% of patients seen in our clinic in 2022 were primary English speakers; of the remaining patients the most commonly spoken languages were Spanish (32%) and Cantonese (8%.) We hypothesize that the need for translation services and cultural factors will shape the telehealth needs of non-English speaking patients, and that remote care solutions will need to be uniquely tailored for these patients. Our research group is currently conducting a follow-up study focusing on Spanish speaking and Cantonese speaking patients, which will yield parallel insights unique to these populations. A second limitation was the absence of patients in the Ideation phase, in which key stakeholders are included to co-design solutions. Findings from our interviews revealed that not every patient might be suitable for telehealth nor might it be desirable by patients to conduct their visit remotely. Our feedback session with clinical faculty aimed to determine clinically-appropriate visits for remote care from the perspective of clinicians, which resulted in a refined screening workflow. Next steps for this study are to test this screening workflow in the clinic, which will include an initial period of high touch evaluation and iteration with both clinicians and patients until a feasible and desirable solution is reached. After these iterations have been made, a longer term pilot will be implemented.

## Conclusions

This study offers an innovative mixed methods approach to designing human-centered surgical telehealth solutions in a safety-net setting. By combining thematic analysis and HCD, this approach yields rich HCD insights that are based on a thematic analysis of the qualitative data. Activities such as brainstorming and co-design invite stakeholder input and place users at the center of solution development, allowing researchers to produce and test a variety of prototypes. We encourage other health clinics, systems and organizations serving medically underserved patient populations to leverage this approach when planning to introduce remote care solutions that can lead to equitable, human-centered surgical care delivery that is tailored to the unique needs and contexts of their stakeholders.

## Supplementary Information

Supplementary Material 1

Supplementary Material 2

Supplementary Material 3

## Data Availability

The datasets developed as a result of this study include transcripts from participant interviews, from which relevant quotes were extracted included in this manuscript. De-identified transcripts are available from the corresponding author upon reasonable request.

## Abbreviations

HCD: “Human-Centered Design”
HMW: “How Might We?”
ZSFG: Zuckerberg San Francisco General Hospital and Trauma Center
