# Supplementary material for: Designing equitable telehealth solutions for outpatient surgical care in a safety-net population: a human-centered design approach
Source: BMC Health Serv Res. 2025 Feb 12;25:236. doi: 10.1186/s12913-025-12215-9 (PMC11817022; doi:10.1186/s12913-025-12215-9)
Supplement: Supplementary file 1 — Supplementary Material 1 [file 12913_2025_12215_MOESM1_ESM.pdf]

Supplementary File 1: COREQ checklist

Consolidated criteria for reporting qualitative studies (COREQ): 32-item checklist

Developed from:

Tong A, Sainsbury P, Craig J. Consolidated criteria for reporting qualitative research (COREQ): a 32-item checklist for interviews and focus groups. International Journal for Quality in Health Care. 2007. Volume 19, Number 6: pp. 349 – 357

Item No	Guide Questions/Description	Reported on Page #
**Domain 1: Research team and reflexivity**		
**Personal Characteristics**		
1. Interviewer/ facilitator	Which author/s conducted the interview or focus group?	Added to page 7
2. Credentials	What were the researcher's credentials? E.g., PhD, MD	Added to page 7
3. Occupation	What was their occupation at the time of the study?	Added to page 7
4. Gender	Was the researcher male or female?	n/a
5. Experience and training	What experience or training did the researcher have?	Added to page 7
**Relationship with participants**		
6. Relationship established	Was a relationship established prior to study commencement?	Added to page 7
7. Participant knowledge of the interviewer	What did the participants know about the researcher? e.g. personal goals, reasons for doing the research?	Added to page 7
8. Interviewer characteristics	What characteristics were reported about the interviewer/facilitator? e.g. Bias, assumptions, reasons and interests in the research topic	n/a
**Domain 2: study design**		
**Theoretical framework**		
9. Methodological orientation and Theory	What methodological orientation was stated to underpin the study? e.g. grounded theory, discourse analysis, ethnography, phenomenology, content analysis	Addressed on page 6
**Participant selection**		

Item No	Guide Questions/Description	Reported on Page #
10. Sampling	How were participants selected? e.g., purposive, convenience, consecutive, snowball	Addressed on page 7
11. Method of approach	How were participants approached? e.g., face-to-face, telephone, mail, email	Added to page 7
12. Sample size	How many participants were in the study?	Addressed on page 9
13. Non-participation Setting	How many people refused to participate or dropped out? Reasons?	n/a – no dropouts, didn't track declines
14. Setting of data collection	Where was the data collected? e.g., home, clinic, workplace	Addressed on page 7
15. Presence of nonparticipants	Was anyone else present besides the participants and researchers?	n/a - no
16. Description of sample	What are the important characteristics of the sample? e.g. demographic data, date	n/a
Data collection		
17. Interview guide	Were questions, prompts, and guides provided by the authors? Was it pilot tested?	Addressed + added clarification to page 7 re: semi-structured interview guide
18. Repeat interviews	Were repeat interviews carried out? If yes, how many?	n/a – no
19. Audio/visual recording	Did the research use audio or visual recording to collect the data?	Addressed on page 7
20. Field notes	Were field notes made during and/or after the interview or focus group?	Addressed on pages 7 and 8
21. Duration	What was the duration of the interviews or focus group?	Addressed on page 7
22. Data saturation	Was data saturation discussed?	Added to page 8
23. Transcripts returned	Were transcripts returned to participants for comment and/or correction?	n/a - no
Domain 3: analysis and findings		
Data analysis		
24. Number of data coders	How many data coders coded the data?	Addressed on page 8

Item No	Guide Questions/Description	Reported on Page #
25. Description of the coding tree	Did the authors provide a description of the coding tree?	n/a
26. Derivation of themes	Were themes identified in advance or derived from the data?	Addressed on page 8
27. Software	What software, if applicable, was used to manage the data?	n/a
28. Participant checking	Did participants provide feedback on the findings?	n/a
**Reporting**		
29. Quotations presented	Were participant quotations presented to illustrate the themes/findings? Was each quotation identified? e.g., participant number	Addressed in table 1 (pages 13-29)
30. Data and findings consistent	Was there consistency between the data presented and the findings?	Addressed on page 32
31. Clarity of major themes	Were major themes clearly presented in the findings?	Addressed in results (pages 10-13) and table 1 (pages 13-29)
32. Clarity of minor themes	Is there a description of diverse cases or a discussion of minor themes?	n/a
