# Supplementary material for: Designing equitable telehealth solutions for outpatient surgical care in a safety-net population: a human-centered design approach
Source: BMC Health Serv Res. 2025 Feb 12;25:236. doi: 10.1186/s12913-025-12215-9 (PMC11817022; doi:10.1186/s12913-025-12215-9)
Supplement: Supplementary file 2 — Supplementary Material 2 [file 12913_2025_12215_MOESM2_ESM.pdf]

April 5th 2022

3M Surgical Clinic
Provider Semi-Structured Interview Guide for Telehealth

Providers will be asked to describe their experience providing care in the 3M Clinic for surgery services. Questions will be open-ended but centered around the following topics:

What is a typical day like for you in clinic?
- What type(s) of patients do you see?
- How many patients do you typically see?
- What do you like about seeing your patients in clinic?
- What are some of the most frustrating parts of providing care in clinic?
- What do you think patients appreciate the most when they see you in clinic?

What has the transition to telehealth been like for you?
- Have you conducted any visits over the phone or video?
- For which kind(s) of patients/visits would it be appropriate or even preferable for you to see remotely?
- What are some of the challenges that you might anticipate in delivering surgical care remotely?
- What are some advantages that you might anticipate in providing care remotely? For you? For your patients?

What would an ideal system of remote care look like for you and your patients in 3M?
- If you could waive a magic wand and design your dream system of telehealth for you and your patients in clinic, what would it look like?
