# Supplementary material for: Designing equitable telehealth solutions for outpatient surgical care in a safety-net population: a human-centered design approach
Source: BMC Health Serv Res. 2025 Feb 12;25:236. doi: 10.1186/s12913-025-12215-9 (PMC11817022; doi:10.1186/s12913-025-12215-9)
Supplement: Supplementary file 3 — Supplementary Material 3 [file 12913_2025_12215_MOESM3_ESM.pdf]

Semi-structured interview guide
for telehealth for 3M general surgery patients

Tell us about your experience attending appointments in the 3M clinic.

- Tell us about your experience attending appointments in person.
  - Has it been easy/difficult for you to attend appointments? Why or why not?
  - How do you feel about going to the hospital for your appointment? What do you like about it? What do you not like about it?
  - What do you like most about seeing your doctors in clinic?
  - What would you change about your in-person visits?

Tell us about your experience using technology in your daily life.

- Do you have reliable access to internet in the places where you spend the most time?
  - If not, where do you go to get internet?
- What do you use technology for the most?
- How comfortable do you feel using technology in your daily life? How much do you rely on technology in your daily life?

Tell us about your experience using your phone/computer/tablet for your appointments.

- Have you used telehealth for primary care, or other types of visits?
- Has it been easy/difficult for you to attend these appointments? Why or why not?
- Where do you prefer to take these appointments? (e.g. home, work, community center, etc.)
- What do you like most about talking to your doctors at [location of choice]? What do you like the least?
- Do you think seeing a surgeon and getting surgical care is different from primary care? Why or why not?
- How do you feel about doing a visit for surgery or after surgery over video/telephone? What do you like about it? What do you not like about it?
- Are there certain types of appointments that you feel more comfortable doing in person? For example: meeting the doctor for the first time, coming back for a follow-up appointment after surgery, etc.? Which of these appointments would you feel comfortable doing using telehealth?
- Do you prefer to use phone or video for your appointments? Why?
- What would make your appointments better?
